# Emerging epigenomic landscapes of pancreatic cancer in the era of precision medicine

**DOI:** 10.1038/s41467-019-11812-7

**Published:** 2019-08-28

**Authors:** Gwen Lomberk, Nelson Dusetti, Juan Iovanna, Raul Urrutia

**Affiliations:** 1 Division of Research, Department of Surgery and the Genomic Sciences and Precision Medicine Center (GSPMC), Medical College of Wisconsin, Milwaukee, WI USA; 2 Centre de Recherche en Cancérologie de Marseille (CRCM), INSERM U1068, CNRS UMR 7258, Aix-Marseille Université and Institut Paoli-Calmettes, Parc Scientifique et Technologique de Luminy, 163 Avenue de Luminy, 13288 Marseille, France

**Keywords:** Cancer genetics, Cancer genomics, Tumour heterogeneity

## Abstract

Genetic studies have advanced our understanding of pancreatic cancer at a mechanistic and translational level. Genetic concepts and tools are increasingly starting to be applied to clinical practice, in particular for precision medicine efforts. However, epigenomics is rapidly emerging as a promising conceptual and methodological paradigm for advancing the knowledge of this disease. More importantly, recent studies have uncovered potentially actionable pathways, which support the prediction that future trials for pancreatic cancer will involve the vigorous testing of epigenomic therapeutics. Thus, epigenomics promises to generate a significant amount of new knowledge of both biological and medical importance.

## Abstract (web summary)

In pancreatic cancer, the epigenomic landscape can strongly impact the disease phenotype. Here, the authors discuss recent advances in our understanding of pancreatic cancer epigenomics, and how this knowledge can integrate with precision medicine approaches in this lethal disease.

## Introduction

Approximately 350,000 people worldwide die every year due to pancreatic ductal adenocarcinoma (PDAC), which makes this cancer one of the most lethal^1^. Despite the extensive research efforts over the past few decades, there has not been a significant improvement in its prognosis, with a 5-year survival rate of <7%, along with a persistent increase in its incidence^1^. Currently, there are no predictive tests for treatment response or prognosis, and therefore, clinicians chose the treatment protocols solely based on the general condition of the patient and stage of the disease. Thus, there is an urgent need to develop methods for predicting whether a patient will benefit from current chemotherapies and which regimen, but even more imperatively to find treatment options for the majority of patients for whom these therapies do not work. Identifying the underlying hallmarks of tumor heterogeneity have become a focus in cancer research^2^, and we believe is the key starting point to determine personalized therapeutic strategies. Most of the actionable targets are directed to pathogenic gene variants or to their tumor-specific neoantigens^3–5^. However, we also need to consider other mechanistic drivers of the cancer phenotype.

**Fig. 1:**
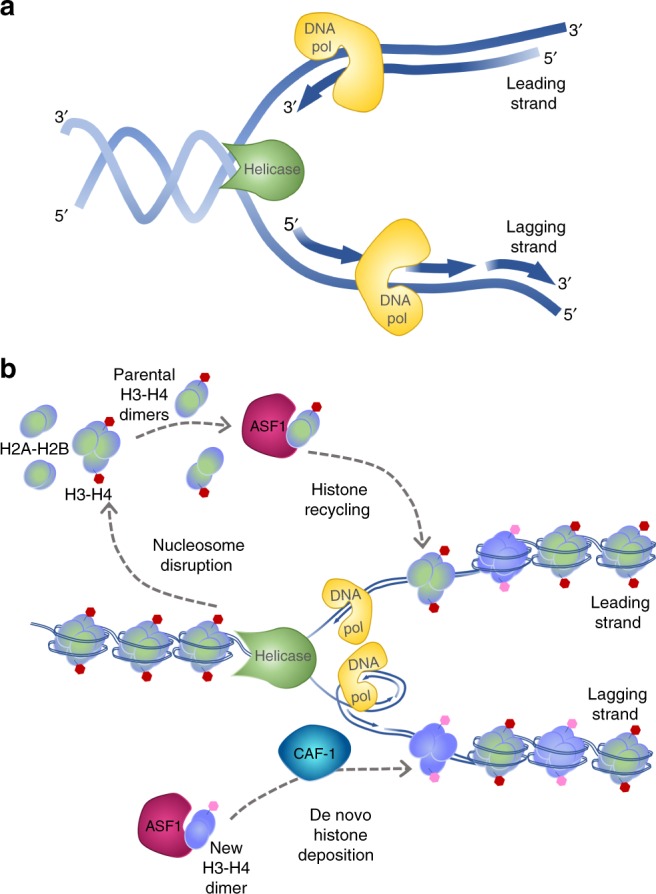
Inheritance at the replication fork. **a** The Naked DNA Paradigm: although the classical semi-conservative model of DNA duplication is useful for explaining Mendelian genetics, the model of DNA in isolation is imperfect for most nuclear processes that require the cooperation of DNA with nuclear proteins, such as inheritance during S-phase, which requires more than just passing a gene to daughter cells. This model simply shows DNA strands unwound at the replication fork by helicase, which is followed by the leading and lagging strands of DNA replication by DNA polymerase. **b** A modern model for inheritance: the more complete model of inheritance, which includes the DNA along with chromatin. At the replication fork, nucleosomes are disrupted to provide access to the DNA polymerase for DNA duplication. These displaced parental histones (shaded green-purple) are reassembled after fork passage with the assistance of chaperone proteins, such as ASF1. In addition, new histones (purple) are required to fully reconstitute chromatin on the two daughter strands, which is also facilitated by chaperone proteins, including ASF1 and CAF-1. New histones acquire marks in accordance to the pattern carried by the parental histones to inherit gene expression states

Since the discovery of the DNA structure as a helix which replicates semiconservatively to pass the genetic material to the progeny, most if not all models only considered naked DNA as the basis of inheritance (Fig. 1a). However, current knowledge strongly supports that the unit of inheritance is chromatin (DNA plus surrounding proteins and RNAs, see Box 1 for definitions) (Fig. 1b)^6^. The consideration of not only DNA, but of chromatin as a whole, with histones and nonhistone proteins, as well as RNAs, is extremely important because environmental cues have the ability to mark areas of chromatin in a manner that modify the regulation of a particular trait. Thus, we must consider that a phenotypic feature is not only given by the genotype, but also by the environmentally modifiable chromatin. Genes are surrounded and regulated by chromatin that senses the environment of cells by writing, reading, and erasing epigenetic marks in tumors and their hosts, in an inheritable manner. The epigenetic landscape was originally coined by Conrad H. Waddington to illustrate the various paths a cell might take toward differentiation^7^. Today, the epigenomic landscape integrates the concepts of the ability of our genome to self-regulate through mechanisms, which assure that the precise set of genes is expressed at the proper level, time, and place to give rise to a particular phenotype and the inheritance of these mechanisms. Therefore, genomics, epigenomics, and nuclear shape cooperate to attain the final cancer phenotype^8^.

Here we discuss emerging and critical evidence that support interactions among genomics, epigenomics, and nuclear structure in PDAC^8^, which together will bring into play a large amount of new potential therapeutic targets and can guide research with both translational and clinical potential. Pancreatic tumors are the consequence of self-reinforcing loops that result in abnormal morphogenesis^9^, for example in PanIN lesions, where oncogenes are the early “game changers”. However, genetic alterations do not explain most aspects of tumor heterogeneity, as well as other cancer hallmarks acquired during tumor promotion and progression^10^. For this purpose, a guiding framework, which we and others have tested for several decades, demonstrates that epigenomics and nuclear structure are key areas necessary for better understanding the behavior of pancreatic cancer and providing new personalized tools for managing this dismal disease.

Box 1 Epigenomic glossary *Epigenetics*: The study of inherited phenotypes through mechanisms that do not involve the coding capacity of the DNA sequence

*Epigenomics*: The study of the collective epigenetic modifications on the entire genome of the cell

*DNA methylation*: The covalent addition of methyl groups to the DNA molecule. Most commonly, this refers to the process of methylation of the five position on the pyrimidine ring of cytosine. As a heritable epigenetic mark, DNA methylation controls gene expression without changing the DNA sequence.

*Chromatin*: The composite of DNA as well as associated proteins and RNAs. Chromatin proteins are divided into histones, which form a structural arrangement to package DNA inside the nucleus, and nonhistones, which play additional regulatory roles.

*Histone*: A highly alkaline nuclear protein that associates with DNA to form the nucleosome and regulate gene expression within chromatin

*Nucleosome*: The basic unit of chromatin which encompasses two turns of DNA wrapped around a set of eight histones, called a histone octamer. An octamer is constituted of two each of the histone proteins H2A, H2B, H3, and H4.

*Histone mark*: A covalent posttranslational modification on a histone protein, such as methylation, phosphorylation, acetylation, ubiquitylation, and sumoylation

*Euchromatin*: Open, permissive chromatin that usually associates with more active gene transcription

*Heterochromatin*: More condensed, tightly packed chromatin with relatively lower gene density and enrichment in repetitive sequences and transposable elements that generally subject to transcriptional silencing

*Writers*: Enzymes that catalyze the addition of a specific posttranslational modification onto DNA or histones

*Readers*: Proteins or protein domains that are recruited to specific epigenetic marks to recognize and bind the mark. Reader domains may be present in writer and eraser enzymes or scaffolds that recruit additional effector proteins.

*Erasers*: Enzymes that catalyze the removal of a specific posttranslational modification from DNA or histones

## The language of the epigenome

As a sensor and transmitter of information, the nucleosome is formed by histones. The deposition or marking of these proteins with specific posttranslational modifications instructs genome regulation by forming areas of euchromatin, which is comprised of open, permissive chromatin, and heterochromatin, referring to more compact, repressive chromatin^11^. These marks are deposited or conversely, removed by molecular machines called chromatin modifying enzymes, which are referred to as writers or erasers of the histone code, respectively, based on their function^8^. Once these marks are established, they are then interpreted by reader complexes to perform a function. The concept of writers, readers, and erasers, however, does not only apply to molecules that modify histones, but also to DNA methylation, which has its own writers (DNMTs), readers (MBDs), and erasers (TETs)^11^. With significant advances in genome-wide methodologies, new humanized animal models (e.g., patient-derived tumor xenografts), as well as the growing popularization of epigenetic models and reagents, we believe this will rapidly translate into more significant advances in the field. Furthermore, mutations in genes that encode MLL2, a writer of the H3K4me3, and KDM6A, an eraser of the H3K27me3 mark, have been found in human pancreatic cancers using genome-wide studies^12^, providing additional rationale to investigate the impact of epigenomic landscapes in this disease.

Accessibility of DNA is facilitated by a family of enzymes called nucleosome remodeling machines, which are able to partially disassemble nucleosomes, exchange histones for variants, assemble nucleosomes, and/or move histone octamers on DNA^8^. These large multimeric complexes bind and hydrolyze ATP to help form and remodel nucleosomes, as well as to move nucleosomes along the DNA track to either expose or cover a region, such as a particular transcription factor binding site. In addition, these nucleosome remodelers frequently interact with chromatin modifying enzymes, which physically integrates the complex to unify nuclear processes, such as gene expression. Interestingly, mutations have also been found in multiple components of nucleosome remodelers in PDAC, including ARID1A among others^12,13^, suggesting a crucial role for nucleosome positioning in the cancer phenotype. Lastly, elegant studies have also described numerous noncoding RNAs that are dysregulated in pancreatic cancer^14^. Since noncoding RNA molecules exert their impact on the epigenome and because of their abundance in exosomes and body fluids, they offer potential therapeutic targets, as well as biomarkers for diagnosis and monitoring disease progression.

## The armor of cell identity: nuclear shape, structure, and dynamics

Pancreatic cells from aggressive forms of cancer have significant alterations in nuclear shape^15–17^. In fact, since the birth of cancer pathology, founding figures in this field, such as Virchow and Lebert, have made the diagnosis of cancer by taking into consideration the appearance of the cell nuclei^18^. The terms of Poikilocytosis or anisokaryosis have been used for almost a century to describe changes in shape and staining pattern of the nuclei. In addition, the extent to which these parameters differ from the normal cell contributes to the pathological score of atypia, which expresses a degree of change compatible with a cell following a pathway toward cancerous transformation. In PDAC, preneoplastic lesions, in particular PanIN 2, are cytologically defined by changes in nuclear shape that initiate at this stage^19^. Thus, we have clinically accepted, at least at the histopathological level, that nuclear changes are present even in precursor lesions, in which there is no certainty as to their path of evolution in the future, whether dormancy or neoplasia. In the past, changes in nuclear shape have been considered a consequence of robust genomic alterations^20^. However, the mutational burden in PanIN 2 is not enough as to completely explain these changes^21^. Thus, it becomes important to discuss how studies on nuclear shape, in particular changes which occur early during cancer initiation, may help to advance pancreatic cancer research. Noteworthy, we should point out that the pathological observations that lead to these descriptions are commonly considered in 2D. However, a comparative 3D representation of the nuclei of a normal cell and its pancreatic cancer counterpart show that distortions in nuclear shape, as well as type and distribution of chromatin, could have significant impact on gene expression patterns, independently of the changes in DNA^22^. For instance, proper duplication of chromatin, the proper spacing of nucleosomes in 3D, as well as the stability of chromosomal territories is responsible for the ultimate identity of a cell type^22–24^. The power of maintaining 3D organization and function of the nucleus as a mechanism of inheritance was first demonstrated by nuclear transplantation experiments, which were recognized with the Nobel Prize in Medicine in 2012^25^. Another important example of the powerful pathophysiological role that changes in nuclear shape, structure, and dynamics of the nuclei can have on the normal phenotype is illustrated by the effects that single point mutations have on lamin genes, which form just the envelope of the nuclei. Morphologically, nuclei from cells carrying lamin A/C mutation are dysmorphic even in the absence of gross genomic aberrations^26^. In fact, the most important change is seen at the level of gene expression, which is compatible with alterations in the shape and volume occupied by chromosomal territories^27^. This disruption ultimately results in inheritable gene expression patterns that are responsible for the Hutchinson–Gilford Progeria Syndrome, a severe form of premature aging^26^. Similarly, we envision that changes in the 3D organization of the pancreatic cancer cell nucleus is both a cause and consequence of transcriptional and posttranscriptional events that determine nuclear functions, a notion of significant mechanistic importance. Therefore, epigenomic studies that aim at defining the contribution of nuclear shape and dynamics will be necessary to reveal the functional impact of these parameters to the cancer phenotype.

## Genomics meets epigenomics to define pancreatic cancer phenotypes

The era of precision medicine has brought a significant increase in the utilization of genomic assays in clinical practice to guide therapeutic decision-making efforts with success^4,28–30^. Given the aggressive biology of PDAC, concern existed for the practical application of such approaches to this disease. In a single-institution study, feasibility has been shown with 336 PDAC patients targeted through deep sequencing of all exons and selected introns of 410 key cancer-associated genes, for which potentially actionable findings were found in 26% of cases^28^. Based on these results, three patients, or ~1% of those tested, received a matched systemic therapy^28^. Expanding the approach to whole-exome sequencing (WES) and RNA sequencing (RNA-seq) in patients with metastatic or locally advanced PDAC, the PancSeq protocol identified therapeutically relevant genomic alterations in 48% and pathogenic or likely pathogenic germline alterations in 18% from a group of 71 patients^4^. As a result of this genomic data, almost 30% underwent a subsequent change in their clinical management^4^. A similar protocol utilizing whole-genome sequencing (WGS) with RNA-seq identified potentially actionable genetic alterations in 30% of patients as part of the Comprehensive Molecular Characterization of Advanced Pancreatic Ductal Adenocarcinoma for Better Treatment Selection trial^29^. In wider multi-institutional efforts, 287 US academic and community practices covering 44 states are participating in the Know Your Tumor program, which has reported on results from the first 640 patients using a combination of genomic, proteomic, and phosphoprotein-based molecular profiling^30^. Actionable genomic alterations were found in 50% of patients, including 27% that were considered highly actionable. The most common of these actionable alterations were in DNA repair genes, such as *BRCA1*, *BRCA2*, or *ATM*, and cell cycle genes, including *CCND1*, *CCND2*, *CCND3*, *CDK4*, and *CDK6*^30^. The same study also reported actionable proteomic alterations, at the exclusion of chemo-predictive markers, in 5% of tumors. Progression-free survival of 4.1 months was significantly longer in a subset of 17 patients who subsequently received matched therapy based on their results compared with 1.9 months for 18 patients with unmatched therapies^30^, suggesting that precision medicine approaches may offer improved outcomes. Whilst precision medicine genomic approaches are increasingly benefiting PDAC patients, there remains the need to extend its power by discovering novel, druggable cancer-associated pathways, using concepts, and methodologies from other disciplines, such as epigenomics, with the goal of expanding the number of actionable targets and benefit a greater number of patients with this disease.

The value of the premise of integrating genomic, transcriptomics, and epigenomic data is supported by data from many laboratories, including ours, which demonstrate that mutational alterations in oncogenes, such as Kras, lead to downstream signaling events that stimulate cell growth in part by modulating histone and DNA modifications through direct regulation of histone proteins, as well as histone and DNA modifying enzymes^31^. Similarly, pancreatic cancer can carry mutations in epigenomic regulators^32^, which by themselves modulate the expression of entire gene expression networks that can support the acquisition of malignant traits. Whilst genetics is critical for pancreatic cancer initiation and early progression, the acquisition of tumor heterogeneity follows specific epigenomic landscapes^33^. In fact, while genomic alterations cannot distinguish between subtypes of pancreatic cancer, epigenetic landscapes dominate subtype-differentiation characteristics^33–35^. Thus, this critical aspect of epigenomic tumor heterogeneity may provide direction in diagnosing and treating this dismal disease.

## Epigenomic landscapes of pancreatic cancer

The Waddington model envisioned the epigenomic landscape as a series of ridges and valleys a cell traverses to acquire epigenomic states on its journey to a final differentiated tissue type^7^. In terms of chromatin regulation, this model is nicely illustrated by work in iPS cells showing that H3K9me3 is an epigenetic mark that antagonizes the acquisition of pluripotency^36,37^. Therefore, we assume that the landscape toward achieving the iPSC state needs to reach a valley, which is low in H3K9me3, which is now possible using novel pharmacological tools. Similar to normal development, epigenetic modifications are also characteristic of disease states, including all cancers, through the progression of precursor lesions to advanced metastatic disease. These epigenetic modifications are thought to play a key role in driving tumor cell heterogeneity. The first attempts for stratifying PDAC tumors were based in genetic mutations. Different exome sequencing studies were carried out that confirmed relatively conserved mutated genes in PDAC (KRAS, TP53, SMAD4, ARID1A, and CDKN2A)^12,13,38^. Nevertheless, limited clinically valuable tumor classification emerged from these thousands of mutations and rearrangements. In addition, other genomic properties such as chromosomal instability index and copy number aberrations showed no association with any PDAC subtype^39^. The lack of genetic support for the major PDAC clinical phenotypes has driven the search for the origin of PDAC heterogeneity in other mechanisms regulated at a post-genetic level.

Initial insight into potential epigenetic differences in PDAC arose from several gene expression analyses of patient cohorts by RNA-seq, which identified signatures of PDAC subtypes with prognostic and biological relevance. Among the first expression-based studies, Collisson et al.^40^ molecularly-defined three subtypes, namely classical, quasi-mesenchymal, and exocrine-like, which displayed differences in clinical outcome as well as therapeutic responses in vitro^40^. Subsequent analyses by Moffitt et al. utilized bioinformatic algorithms to separate gene expression patterns arising from tumor, stroma, and normal tissue^41^. As a result of this “virtual microdissection”, these subtypes were distilled to a classical and a basal-like tumor-specific signature^41^. Whilst the prior “quasi-mesenchymal” subtype seemed to comprise a mixture of gene expression patterns from the basal-like tumors and stromal components, the “exocrine-like” subtype appeared to have gene expression signatures indistinguishable from adjacent normal tissue, suggesting an issue of contamination in the prior classification^41^. Integrated analysis of both genomic and transcriptomic data by the Australian Pancreatic Cancer Genome Initiative identified squamous, pancreatic progenitor, aberrantly differentiated endocrine exocrine (ADEX), and immunogenic subtypes^42^. Examination of these four subtypes suggests that the squamous, pancreatic progenitor, and ADEX match the quasi-mesenchymal, classical, and exocrine-like subtypes, respectively, while the immunogenic subtype was newly defined and appears to reflect high immune infiltrates. The Cancer Genome Atlas consortium analyses supported the two-subtype classification model of classical and basal-like, demonstrating that the exocrine-like/ADEX and immunogenic subtypes were likely the result of non-tumor contaminants^43^. Thus, part of the heterogeneity observed among PDAC patients can be explained by the classification into different subgroups of clinical outcome and therapeutic responses.

To gain a clear understanding of the epigenomic landscapes of pancreatic cancer, it has been important for the field to integrate data from several new methodologies directed to achieve not only descriptive, but also mechanistic power. Our studies have demonstrated by a multi-omic approach (RNA-Seq, miRNA-Seq, Exome-Seq and methylation, and SNP chips) in xenografts derived from patients, that the basal and classical PDAC subtypes could be classified by specific alterations in their DNA methylation pattern^39^. These results indicate that the main PDAC outcome phenotypes are established through the epigenomic landscape rather than its genetic alterations. For example, the DNA methylation patterns of several effectors and inhibitors from the WNT signaling network are altered in the basal subtype. However, in the classical samples, small molecular transporters, such as the glutamine SLC1A1 transporter, and cholesterol transporters, like NPC1L1, are overexpressed as a consequence of DNA hypomethylation. Our most recent work implemented an experimental design to provide a comprehensive view of genomic regulatory elements, which included the integration of ChIP-seq from five histone marks along with RNA-seq and DNA methylation datasets^33^. Histone modifications associated with distinct transcriptional regulatory outcomes were selected, such as H3K4me3 for active promoters, H3K27ac for active enhancers and promoters, H3K4me1 for active and poised enhancers, H3K9me3 for heterochromatin, and H3K27me3 for Polycomb-repressed regions. In concordance with Nicolle et al.^39^, Lomberk et al. described the classical subtype to be associated with pancreatic morphogenesis (e.g., PDX1, BMP2, GATA6, and SHH) and metabolic processes (e.g., HKDC1 and FBP1)^33^. Functional analysis of the basal subtype found genes related to oncogenic signaling pathways (e.g., ErbB/EGFR, PI3K-AKT, Hippo, and Wnt), EMT (TGFβ pathway), and deregulation of cell differentiation, proliferation, and apoptosis (e.g., YAP1, HEY1, MYC, and E2F7), reflecting the more aggressive nature of the basal tumor subtype.

**Fig. 2:**
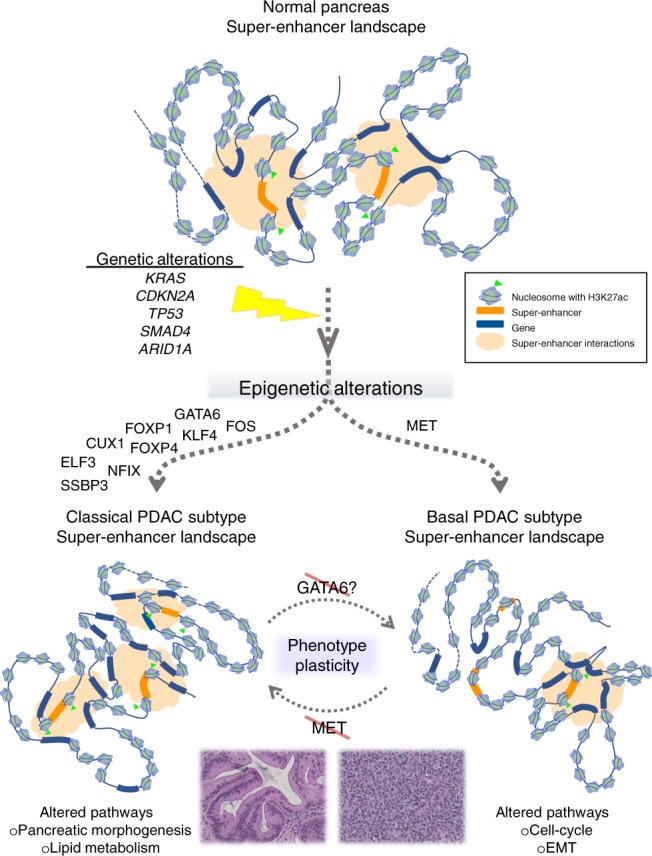
Epigenomic landscapes explain the progression of PDAC into two subtypes. While PDAC initiates with certain genetic alterations, including *KRAS*, *CDKN2A*, *TP53*, and others as indicated, mounting evidence reveals that the subsequent establishment of the classical and basal subtypes is a consequence of epigenetic alterations, which include specific super-enhancers driven by distinct upstream regulators to result in altered gene expression networks that define each subtype^33^. Experimental data from knockdown of MET in the basal subtype substantiates the potential conversion to the classical subtype, suggesting the possibility of phenotype plasticity^33^. Similarly, it may be feasible to convert the classical into the basal subtype through targeting upstream transcription factors, such as GATA6

Importantly, our multi-parametric integrative analyses of the datasets revealed that super-enhancers better reflect the heterogeneity of the different PDAC subtypes (Fig. 2). Specific super-enhancers were identified in the classical tumors, which seemed to be influenced by at least nine different transcription factors, including GATA6, FOS, FOXP1, FOXP4, KLF4, ELF3, NFIX, CUX1, and SSBP3. Notably, high GATA6 levels have been consistently associated with the classical subtype^29,33,40–42^. Furthermore, these enhancer-associated transcription factors appear to amplify their function through regulating other transcription factors that regulate distinct functions, such as metabolic networks implicated in the classical subtype^33^. On the other hand, the regulation of basal-specific super-enhancers was associated with the hepatocyte growth factor receptor (MET) rather than a specific transcription factor^33^. Knockdown of MET stimulated the conversion of the basal into the classical “molecular phenotype”. Therefore, there exists the potential to induce certain types of plasticity between both phenotypes, which opens the opportunity for experimental therapeutics. Several transcription factors were identified downstream of MET that are involved in proliferation (MYC, MYBL1, and E2F1) and EMT (SNAI2), which should play key roles in the development of basal tumors, thus providing additional information of high pathobiological value. In a more recent study, the squamous or more basal-like human cell line, BxPC3, which expresses the oncogenic ∆N form of TP63 (∆Np63) shown in human primary PDAC samples of this subtype, underwent reprogramming of enhancers associated with the squamous gene signature upon CRISPR/Cas9-mediated deletion of this transcription factor^35^. These studies highlight the plasticity of subtypes based on enhancer landscapes and identify potential vulnerabilities for therapeutic targeting.

From the biological point of view, these studies advance our understanding of how gene expression and morphogenetic pathways, which are epigenetically regulated, result in types of cancers with apparent dissimilar outcomes. Noteworthy, this topic will take us to consider the current validity of preexisting paradigms and their adaptations. For instance, “dedifferentiation”, which has been associated with malignancy, has been promoted for decades to be a hallmark of cancer^44^. However, similar to normal tissue, basal and classical pancreatic cancer subtypes follow distinct molecular differentiation patterns in a reproducible and inheritable pattern, through distinct epigenomic landscapes. It is provocative to consider whether we are born with both, a predictable normal and a similarly predictable cancer phenotype. Analyses of our RNA-Seq datasets^33^, indicates a significant correlation with the two subtypes described by Moffitt et al.^41^, suggesting that these subtypes are stable as to at least maintain their pattern of gene expression, even when passage through mice.

The second conclusion is that these subtypes cannot be distinguished on the basis of pathogenic somatic cancer-associated variants. In fact, we propose that the study of PDAC tumor subtypes within the pipeline of a Cancer Precision Medicine Clinic must incorporate RNA-Seq. Histone marks, DNA methylation, as well as large and small noncoding RNAs, all components of the epigenomic orchestra, are more discriminative than genetic tools as it relates to tumor phenotype. The molecular data that results from the full integrative analyses of enhancers and super-enhancers provide transcriptionally active areas that are likely to convey the subtype-specific gene expression pattern for the tumors. For instance, classical tumors, with a propensity for the increase of specific super-enhancers, are more likely to follow a landscape that is marked by high levels of H3K27ac, demonstrating a dominant effect of these genomic regulatory regions for maintaining the phenotype of these tumors, while C-Met was predominantly associated with the landscape of the basal phenotypes (Fig. 2). The amount of information, however, that these datasets^33^ provides is far richer than what can be described here, but offers a foundation for future diagnoses, prognoses, and treatments, as well as a plan for additional mechanistic experiments.

The underlying genetic alterations of PDAC are unable to distinguish subtypes as mentioned above, but rather epigenetic landscapes are able to identify subtypes. Similarly, within a single untreated PDAC patient, the genetics of metastatic lesions is not discernable from the primary tumor^45^. Utilizing samples of matched primary and metastatic PDAC lesions collected by rapid autopsy, McDonald et al.^34^ performed ChIP-seq on a series of histone marks for heterochromatin (H3K9me2, H3K9me3, and H3K27me3) and euchromatin (H3K27ac and H3K36me3) to demonstrate that global epigenetic reprogramming is also responsible for the heterogeneity of tumors in each individual patient^34^. Specifically, the epigenetic landscapes of both H3K9me2 and DNA methylation were found to undergo global remodeling within heterochromatin domains called LOCKs (large organized chromatin K9-modified), as well as localized chromatin changes in H3K27ac and H3K36me3 of differentially expressed genes.

The important contribution of enhancers to the epigenetic landscape of PDAC during discrete stages of disease progression has also been supported in studies from a genetically-engineered mouse model^46^. Through the use of organoids grown from cells isolated from the KC and KPC mouse models at different stages of PDAC, Roe et al.^46^ found that the transition to metastatic potential is accompanied by alterations of the enhancer landscape with notable and consistent changes in global H3K27ac enrichment^46^. Interestingly, the authors identified FOXA1 as a main driver of this epigenetic landscape, promoting enhancer activation and metastasis in vivo. While our analyses from human tumors did not identify FOXA1 as an upstream regulatory node^33^, FOXA1 upregulation has been found in human samples to be a prevalent feature in more advanced primary tumors and metastasis^41^. Notably, in human PDAC cells, FOXA1 has been recently shown to be regulated by GATA6^47^, which was identified as a central node in our studies^33^. Further consideration of PDAC subtypes^42^ revealed that FOXA1 upregulation is associated with the classical rather than basal subtype. Thus, the acquisition of an enhancer-mediated epigenetic landscape seems to be a feature associated with molecular drivers of the classical subtype, in both mouse models of this disease^46^ and PDAC patients^33^.

The contribution of the tumor microenvironment is well-established in cancer, and in particular with the stroma present in pancreatic cancer. In a study by Sherman et al.^48^, the influence of the pancreatic cancer stroma to dramatically alter survival of cultured PDAC cell lines under conditions of growth challenge was associated with alterations in the epigenetic landscape of the tumor cell^48^. In particular, factors secreted from stromal cells were able to induce a rapid transcriptional response in gene networks involved in core metabolic pathways in the TCA cycle, anabolic metabolism, and cell growth. Taking advantage of the distinct genomes of the implanted human tumor and mouse stromal compartments present in patient-derived xenograft models, we have been able to provide genome-wide analysis of signaling pathways involved in tumor-stromal cross-talk, which indicated that the stroma is closely associated with the phenotype of the tumor^39^. Furthermore, when tumor and stromal features are considered, the classification system can be integrated to reflect the relative contributions of various components into five subtypes, namely pure basal-like, stroma activated, desmoplastic, pure classical, and immune classical^49^. Interestingly, this analysis has highlighted differences in tumor subtype-specific roles of the tumor microenvironment for prognosis, such that basal-like tumors fare worse without a stromal component (e.g., “pure basal-like”), whereas pure classical tumors are associated with the best survival rates. This suggests a protective role of the stroma in the basal-like subtype and an opposite, tumor-promoting function in the classical subtype. Thus, the challenges facing the therapeutic targeting of the tumor microenvironment may also reflect the impact of this compartment on the epigenetic landscape of the pancreatic tumor and vice versa.

Analyses of both the evolution and innovation in the field of precision medicine, combined with the rapid advances in data-driven health care delivery centers, suggest that the field of cancer precision medicine may identify cancers more as molecular entities rather than by their organ of origin^50,51^. This concept has significant implications for prevention, diagnosis, prognosis, therapeutics, treatment monitoring, and genetic counseling. For instance, the implementation of precision medicine for pancreatic cancer can no longer be practiced without the assembly of a team-science approach nor can it be simply addressed using a single assay modality. The of use gene exome panels, WES, and WGS to the search for genetic predisposition and potentially actionable genomic variants, including those that predict response to tumor-directed therapy and pharmacogenomics are among the precision medicine assays that have shown clinical utility^52,53^. The latter can affect the levels and/or efficacy of pain and anti-clotting medications, impact the quality of life, and has become reimbursable by many systems. RNA-seq is more useful for the identification of subtypes in treatment-naïve tumors and as described in a previous section, provide useful information when appropriately analyzed^40–43,49^. Epigenomic panels for nucleic acid modifications and histone-based assays have the advantage of being rapidly applicable since they are based on hybridization or antibodies, which can be directly applied to tumor diagnoses from biopsy material. We predict that assays based on these types of markers will soon be part of the arsenal of the molecular pathologist at the bedside. Companion assays for histone-based epigenomic therapies are being developed but not yet applied to pancreatic cancer^54^. Epigenomic assays are increasingly becoming more miniaturized and assisted by robotics and microfluidics^55,56^, which can help to expand the repertoire of assays being developed. Epigenomics is at the frontier of precision medicine, where new knowledge is rapidly emerging and the translation to the clinic is likely to arrive earlier than expected.

## Harnessing the value of epigenomic inhibitors in pancreatic cancer

Early studies on targeting the function of different components of the epigenome, which bear pharmacological importance, focused on transcription factors such as p53^57^. This approach is attractive since transcription factors hold high specificity in recruiting epigenomic regulators to distinct areas of the genome. However, transcription factors are, for the most part, devoid of enzymatic activity rendering these molecules difficult to be targeted by active site inhibitors^58^. On the other hand, the targeting of epigenomic regulators, which have enzymatic activities, are by nature less specific since those complexes can be recruited by a larger number of transcription factors to genes that can have, in many instances, opposite functions (tumor suppression vs. tumor promotion). In addition, it is important to discuss biases in the current design and testing of epigenomic pathways. The first challenge is that many studies have been designed to demonstrate severe inhibition of cell growth by these types of drugs in short-term culture assays, which for the most part only test the cytotoxic effects of these drugs. A better understanding of epigenetic processes and the incorporation of this knowledge in the design of therapeutic strategies has stimulated the use of epigenomic-based inhibitors in combinations or as chemosensitizers through reprogramming resistant tumors^59^. Thus, based on this trend, we anticipate that the design, synthesis, testing, and use of epigenomic inhibitors in the clinic will continue expanding at an accelerated rate.

A detailed analysis of our most recent studies indicates that different subgroups of tumors are clustered according to their levels of distinct histone marks^33^. In fact, for the most part, we can identify at least two or three subgroups of individuals based on these criteria. However, we currently do not know as whether the manipulation in the levels of these marks, through targeting the enzymatic complexes that either write or erase them, has any impact on the malignant properties of any of these particular tumors. Epigenomic pharmacology is an actively expanding field, which provides a growing arsenal of potential therapeutics to evaluate for the treatment of pancreatic cancer (reviewed in ref. ^60^). The potential of these pathways to regulate pancreatic cancer-associated properties has been, for a long time, an underrepresented area of research. However, emerging data using genetic and/or pharmacological tools support the ability of some of these enzymatic complexes to regulate distinct aspects of the pancreatic cancer phenotype, with the caveat that the data primarily remains in the preclinical testing phases of research.

The understanding of PDAC heterogeneity and the underlying epigenetic mechanisms^33^ offers the possibility to identify new potential therapies with specific targets based on tumor phenotype. On the other hand, the pancreatic tumor cells continually adapt to metabolic pressures for survival in a particular microenvironment^34^. The extensive PDAC stroma exhibits low vascularization contributing to hypoxia and nutrient deprivation with subtype-specific characteristics. From our work, we proposed an approach to target the epigenetically deregulated cholesterol transport NPC1L1 by its well-known inhibitor ezetimibe, a safe drug that is effectively used against hypercholesterolemia^61^. High cholesterol intake has been reported to increase the risk of pancreatic cancer^62^ and identified as a key metabolic pathway during PDAC progression^63^. However, targeting this cholesterol transporter has not been previously explored. Treatment of both, organoids and xenografts with ezetimibe significantly decreased PDAC growth, indicating NPC1L1 is a highly effective therapeutic target^39^. This proof-of-concept supports that epigenetic characterization of aberrantly regulated pathways offers a promising strategy for identifying targets for PDAC.

In a similar manner, other epigenetically deregulated pathways present potentially druggable candidates. For example, the inhibition of the WNT pathway in basal tumors is a promising approach as there are several WNT signaling-targeted therapeutics in preclinical phases or clinical trials for the treatment of cancers associated with WNT alterations (e.g., vantictumab, cirmtuzumab, and rosmantuzumab)^64^. Furthermore, we find that growth of pancreatic tumors with particular profiles, such as high MYC expression, can be effectively inhibited with select agents, namely inhibitors of the BET family of chromatin adapters^65^. While the MYC molecule itself is challenging as a pharmacological target, the discovery that BET inhibitors result in the effective reduction of MYC transcript and protein levels^66^ has highlighted the possibility of utilizing epigenetic pharmacology in cancer therapy to circumvent molecules unamenable to direct targeting.

In addition, new strategies could focus on not only the difficult mission of eliminating all cancer cells, but also converting the worst outcome phenotype to one with a better prognosis. With this aim, based on our latest study, which associated C-Met with the epigenomic landscape of the more aggressive, basal PDAC subtype, we demonstrated that knockdown of C-Met by small interfering RNA (siRNA) modified the regulation of super-enhancers and consequently, shifted tumors from a more aggressive toward a more benign tumor phenotype (Fig. 2)^33^. Interestingly, in other cancers such as non-small cell lung cancer and hepatocellular carcinoma, anti-MET therapy with monoclonal antibodies and small-molecule inhibitors has been used already in clinical trials with promising efficacy and a low toxicity profile^67^. Therefore, this study encourages future therapies targeting the MET pathway, alone or in combination with standard therapies. Furthermore, these results are the first evidence that it is possible to modify the tumor phenotype; thus, we can hypothesize that it is also probable to alter the pharmaco-types and resensitize a resistant cell to particular therapies. Lastly, an important observation that bears relevance to targeting tumors with precision is the fact that some tumors bear distinct combinations of marks, raising the possibility that the epigenome of each of these tumors is rather “individualized” and unique targeting strategies may be required.

## Conclusions and forward perspective

Epigenetics was methodologically born from studies at the single gene level of a few loci. Of importance in the field of pancreatic cancer, for instance, high levels of H3K27me3 and DNA methylation have been found to play a prominent role in silencing of the *p16INK4a* tumor suppressor gene^68,69^. With the sequencing of the human genome^70^ and birth of microarray technologies, chromatin-based (e.g., ChIP-chip) and methylation arrays extended the knowledge to a genome-wide level^71^, giving rise to epigenomics. Epigenomics is a part of the system, namely the cells, tissues, organisms, populations, and even tumors, and thus, when combined with other data, allows one to develop a systems biology model. The value of collecting and integrating multiple types of data from the system allows compensation for absent or unreliable data from a single source. On the other hand, the bioinformatics-based modeling of data derived from many epigenomic methodologies makes significant assumptions (e.g., cutoffs), often uses bioinformatics-based definitions with uncertain biological equivalence and is not easy to integrate^72^. The results acquired by state-of-the-art methodologies, such as those used in our recent study^33^, are developed to visualize their output on a two-dimensional map of the human genome (e.g., UCSD Genome Browser). Through the rational, combined use of these methodologies, significant information can be obtained on gene regulatory regions that are responsible of a particular cancer-associated trait. In fact, numerous large-scale epigenome mapping projects (e.g., ENCODE^73,74^ and AHEAD^75,76^) of DNA methylation, histone modifications, chromosome interaction analyses, transcription factor binding sites, among others have markedly increased the datasets available from various normal and diseased cell types^72^. However, substantial challenges remain regarding data analyses, integration, management, and security.

Emerging methods for better data integration have been focused on filling the gap that exists between generating large volumes of data and our understanding of biology to reproduce the complexity within biological systems. The ability to model the association between phenotypic outcome (i.e., biological complexity) and variations identified by high-throughput multi-omics will advance our comprehension of underlying mechanisms and/or causal relationships of disease architecture and etiology. Importantly, data quality, data scale or dimensionality, and potential confounding of the data need to be carefully considered for each individual data type before integration to prevent downstream issues with the analysis. Currently, two main approaches are utilized for data integration: multi-staged analysis and meta-dimensional analysis^77^. Multi-staged analysis integrates data through the use of a stepwise or hierarchical analysis approach; while meta-dimensional analysis builds a multivariate model associated with a given outcome by simultaneously combining multiple different data types. These models of data integration open the exciting possibilities to explore new scientific questions. It has become increasingly clear that no single analysis approach will be advantageous for all investigations. Thus, the continual evolution of bioinformatics approaches for big data to develop an extensive analysis “toolbox” will be essential for future discoveries and interpretations in the field.

On the methodological front, new trends involve the development of organs-on-chip (OOC) models^78^ and humanized mice^79^, as well as miniaturization and automatization^80^. For instance, studies of intratumor heterogeneity will require single-cell sequencing technologies that are rapidly emerging for genomic, transcriptomic, and epigenomic profiles^81^. The feasibility and limitations of this powerful technological advance have been influenced by the development of combinatorial indexing methods for high-throughput genome-wide sequencing, as well as improved techniques for recovery yields from a single cell or limited cell number. In addition, to push forward the validity of our guiding model^8^, methodologies that provide insights into the 3D structure of the nucleus will be fundamental. Since the genome does not function solely in a sequential manner, but rather organized in 3D space, we will only gain a thorough understanding of genome functionality in disease states, such as pancreatic cancer, if we have tools to map contacts among remotely located genomic elements, which regulate each other. Among these methodologies are the original chromosome conformation capture (3C) technology introduced in 2002, which is a “one-to-one” method to find contact frequencies between chosen pairs of genomic sequences, followed by the development of higher-throughput modifications of the 3C technique, including the “one-to-all” 4C or circularized 3C method, the “many-to-many” 5C or 3C carbon copy approach, and finally, the “all-to-all” Hi-C introduced in 2009, which combines chromosome capture with next-generation sequencing (NGS) to obtain whole-genome contact maps^82^. This technique has been effectively used to start understanding noncoding regions of the genome, which may harbor cancer-driving mutations. For instance, Hi-C has revealed that a recurrently mutated cis-regulatory element in colon cancer interacts with the *ETV1* promoter, thereby affecting gene expression^83^. In fact, many of the single nucleotide polymorphisms (SNPs) linked to cancer risk identified by large-scale genome-wide association studies (GWAS) map to noncoding regions that are several hundred kilobases from the nearest protein-coding genes^84^. The utilization of capture-based Hi-C technologies has provided functional insight for the first time into high risk loci in cancers, such as breast, prostate, and colon^85–87^. 3C-based techniques have been also used to gain functional information regarding at least one common susceptibility locus for pancreatic cancer in a 610 kb gene desert on chr13q22.1^88^. Furthermore, this 3C technology has been combined with ChIP, known as chromatin interaction analysis by paired-end tag sequencing or ChIA-PET, to study contacts between genomic sequences bound by a protein of interest. For instance, long-range chromatin interactions associated with the androgen receptor and its collaborative transcription factor, erythroblast transformation-specific related gene, ERG, that underlie coordinated gene expression in prostate cancer were mapped via ChIA-PET^89^. Notably, these regions were also highly enriched in SNPs associated with prostate cancer. As these various 3C-derived methods are refined and NGS progressively increases resolution, we will expand our knowledge of novel properties of genome structure along with their functional implications, and we expect these technologies to open the next frontier in pancreatic cancer research.

In summary, we conclude that an even more accurate view of pancreatic cancer will be achieved when cancer-associated pathways are considered as a combined alteration in “genomic-epigenomic-and-nuclear” structure. In this organ, it appears that early preneoplastic lesions require only few mutations to trigger a process of abnormal organogenesis via self-reinforcing pathological loops^9^. During progression, epigenomic landscapes, defined by the differential acquisition of enhancers and super-enhancers, appear to be necessary to maintain inheritable, cancer-associated gene expressions patterns that support the heterogeneous differentiation of human pancreatic cancer tumors^33–35^. This has provided unique insights into an arsenal of novel, potentially actionable pathways, which were not previously obtained through genomic analyses, supporting the notion that effective future therapeutic regimens for PDAC will require precision medicine approaches that include epigenomic targets.
